# Protein Mass Fingerprinting and Antioxidant Power of Hemp Seeds in Relation to Plant Cultivar and Environment

**DOI:** 10.3390/plants12040782

**Published:** 2023-02-09

**Authors:** Chiara Cattaneo, Annalisa Givonetti, Maria Cavaletto

**Affiliations:** 1 Department of Science and Technological Innovation, DiSIT, University of Eastern Piedmont, 13100 Vercelli, Italy; 2 Department for Sustainable Development and Ecological Transition, DiSSTE, University of Eastern Piedmont, 13100 Vercelli, Italy

**Keywords:** hemp, seed, proteins, albumins, MALDI-TOF, SDS-PAGE, antioxidants

## Abstract

*Cannabis sativa* (hemp) seeds are considered a functional food for their favorable contents of essential fatty acids, proteins and antioxidants. Beyond phenolics and carotenoids, the bioactivity of proteins has recently been investigated. However, plant genotype and environmental conditions can affect quantity and quality of macronutrients and phytochemicals in seeds, influencing their nutraceutical properties. In this study, the effects of plant variety and seed origin on the protein profile and antioxidant activity of hemp seeds were evaluated. Seeds from two cultivars, Secuieni Jubileu and Finola, were harvested from a mountain field located in Italy and compared with reference seeds used for sowing. Albumin and globulin extracts were obtained using the Osborne method and their antioxidant power was assayed (DPPH and ABTS methods). A matrix-assisted laser desorption ionization time-of-flight (MALDI-TOF) mass spectrometry method was developed for protein fingerprinting analysis. Albumins from seeds of the mountain site showed higher radical scavenging activity and compounds of lower molecular weight than reference seeds, suggesting a role of proteins in the observed bioactivity. The MALDI-TOF method discriminated samples according to origin and variety, highlighting changes in the protein profile and identifying signals which could be used as markers of hemp cultivars.

## 1. Introduction

*Cannabis sativa* is an annual herbaceous plant which has encountered an alternating fate. In the past, it has played an important role in food, fiber and medicine production [1], but its cultivation declined by the middle of the 20th century. The reasons can be found in the availability of more convenient sources of textile fibers, such as cotton or the development of synthetic fibers, and in the diffusion of cultivars with psychotropic effects, which were banned by the law. Therefore, legal use of *Cannabis sativa* is limited to varieties also known as “industrial hemp”, which are characterized by a content of tetrahydrocannabinol (THC) as low as 0.2%, which defines the upper legal limit to be cultivated in Europe. After years of oblivion, in recent times, hemp has gained a second life thanks to its versatility: beyond the use of fibers for the textile industry, its biomass can be exploited for the production of bio-plastics, biofuels or biochar for soil enrichment. Currently (January 2023), the European Plant Variety Database (European Commission EU Plant Variety Database) includes 100 hemp cultivars allowed for industrial cultivation, demonstrating the increasing effort in breeding plants for multi-purpose production. Moreover, hemp seeds, which have been treated as by-products of *C. sativa* cultivation for a long time, are now considered a sort of “superfood” due to the discovery of a favorable content in omega-3 and omega-6 polyunsaturated fatty acids (PUFA) and high-quality proteins. Therefore, hemp seeds are part of the increasing number of “unusual” seeds that have been included in the human diet as a source of functional foods, since they have been recognized to provide a positive effect on human health, including the prevention and management of non-communicable diseases such as diabetes, cardiovascular diseases, Alzheimer’s disease and cancer. Hemp seeds can be consumed whole or dehulled (hemp hearts), or crushed for the production of hemp flours. Due to their richness in valuable fatty acids, the lipid fraction can be recovered from cold-pressed seeds and commercialized as hemp seed oil. The remaining residue is the “hemp cake”: formerly considered as a by-product, this is a protein-rich resource and can be exploited as animal feed, or as a food supplement. Hemp seeds contain about 25–35% lipids, 20–25% proteins and 20–30% carbohydrates, with 10% of insoluble fibers [2]. The fatty acid profile of hemp seeds is particularly favorable to human health, due to the high amount of PUFA (80% of the total content) mainly represented by the essential fatty acids (EFA) linoleic acid (LA) and alpha linolenic acid (ALA). Moreover, the content of gamma linolenic acid (GLA) and stearidonic acid (SDA) can reduce the formation of inflammatory molecules. The protein fraction of hemp seeds is mainly represented by storage proteins 11S globulin (edestin, 60–80%), 2S albumin (13%) and 7S vicilin-like protein (5%) [3]. Hemp seeds are also a source of minerals such as phosphorus, potassium, magnesium and calcium, and molecules with antioxidant activity, such as tocopherols and lignan derivatives [4]. Reactive oxygen species (ROS) are a by-product of oxidative metabolism and cause inflammatory diseases and aging processes if their production is not counterbalanced by antioxidant molecules. ROS such as hydrogen peroxide, superoxide anion and hydroxyl radical can irreversibly damage lipids, proteins, carbohydrates and nucleic acids, ultimately leading to cell death [5,6]. If the endogenous defense systems consisting of antioxidant enzymes (e.g., catalase, superoxide dismutase, glutathione peroxidase) and metal-binding proteins cannot efficiently mitigate the oxidative imbalance, further help can be provided by external molecules. In this respect, foods of plant origin represent a good source of antioxidants with potentially no harmful side effects. The more acknowledged antioxidants include products of the plant secondary metabolism, such as phenolic compounds (e.g., flavonoids) and carotenoids. However, an increasing number of studies report the role of proteins and peptides not only as nutrients but also as bioactive molecules with antioxidant, antihypertensive, hypocholesterolemic, antidiabetic and other interesting properties. The type and level of activity result from a combination of factors: the sequence of amino acids, the degree of hydrolysis and the chosen protease to obtain the final hydrolysate. Therefore, the search for “hidden treasures” in plant proteins has started, with the aim of turning by-products into new value-added goods, possibly with nutraceutical function. The main sources of bioactive peptides are represented by legumes such as soybeans, peas, lentils and lupins, and also cereals (e.g., rice) and pseudocereals (e.g., amaranth and quinoa). Regarding hemp seeds, the presence of bioactive peptides with antioxidant, ACE inhibitory, hypocholesterolemic and dipeptidyl peptidase IV (DPP-IV) inhibitory action has been reported so far [7,8,9,10]. Nonetheless, variations in lipid, protein and carbohydrate contents of hempseed can be related to plant genotype, and environmental conditions can affect the phytochemical profile, influencing the content of phenolics and antioxidant activity [3,11,12]. Matrix-assisted laser desorption ionization time-of-flight mass spectrometry (MALDI-TOF MS) is an analytical technique that has been successfully used for the analysis of foods, microorganisms and clinical samples, with the aim of identifying unique mass spectra, also known as “fingerprints”, for the evaluation of food authenticity, discovery of clinical biomarkers or microorganism identification. Some applicative examples of MALDI-TOF MS protein fingerprinting include the determination of food origin (e.g., kimchi, milk, feta cheese), the monitoring of protein modification during a bioprocess (e.g., malting) and the discrimination of pollens belonging to different populations [13,14,15,16,17]. The strength of this technique is in the quick sample preparation, generation of reproducible results and relatively easy interpretation of mass spectra, compared to other mass spectrometry techniques.

In this study, the protein profile and antioxidant activity of seeds produced by two “industrial hemp” (i.e., with THC levels below 0.2%) varieties, Secuieni Jubileu (monoecious) and Finola (dioecious), were investigated. Seeds that were harvested during the 2018 (Finola) and 2019 (Secuieni J.) growing seasons from an experimental field located in mountain site of Piedmont, Italy, were compared with seeds that were used as starting material for sowing (denoted as “certified seeds”). The Osborne fractionation method was chosen to extract proteins with different solubility since it works with mild conditions that do not denature proteins, allowing the study of hemp seed storage proteins in their native form.

The objectives of this study were as follows:

− To assess effects of the climatic conditions of the growing area on the protein profile and antioxidant activity (determined by means of the DPPH and ABTS assays) of the water- and salt-soluble fractions obtained from the two hemp varieties;
− To compare seed protein extracts by MALDI-TOF MS to highlight differences related to plant variety and origin, providing a rapid method for protein analysis.

## 2. Results

### 2.1. Seed Weight and Weather Parameters

Hemp seeds were sown at the mountain site of Viganella (Piedmont, Italy) during the last two weeks of May of the years 2018 (Finola) and 2019 (Secuieni J.). Monthly rainfall and temperatures registered during the years 2018 and 2019 by the meteorological sensor nearest to the experimental site are presented in Supplementary Table S1. During the period from April to September, the year 2018 showed a higher total amount of rainfall than 2019 (715 vs. 619.8 mm). The year 2019 had more precipitation during the months of plant growth (June and July) and was cooler than 2018 (20.1 °C vs. 24 °C maximum temperature), although it must be considered that temperature data of June were not available for the year 2019.

Seeds of Finola and Secuieni J. varieties that were obtained from the experimental field (harvested seeds) were compared with the seeds used for the sowing (certified seeds). The weight distribution of 50 seeds from Finola and Secuieni J. varieties, certified or obtained from the experimental field, is represented in the box plot of Figure S1. Concerning certified seeds, those of Secuieni J. show higher weight than Finola seeds (Table 1). However, this difference is not detected in seeds from the experimental field, highlighting an environmental effect on this parameter. In fact, harvested seeds of both varieties underwent a weight decrease compared to certified seeds (Finola −23%, Secuieni J. −44%).

### 2.2. Antioxidant Activity Estimation by In Vitro Assays

Due to the different nature of phytochemicals in plant extracts, it is important to consider different methods to evaluate the antioxidant activity. For this reason, two assays were chosen: the DPPH and ABTS methods. The radical scavenging capacity is an expression of the antioxidant activity of the sample extract. In the presence of such activity, the color of the stable DPPH and ABTS radicals diminishes proportionally to the concentration of antioxidants in the sample.

ABTS, in the presence of potassium persulfate, forms a free radical soluble in water and organic solvents, making it suitable for testing lipophilic and hydrophilic compounds. In the presence of a hydrogen donor, the greenish ABTS**⸱**^+^ radical form is converted into the colorless form of ABTS. The radical scavenging activity of hempseed extracts determined using ABTS assay is expressed as μMTE (Trolox equivalents)/mg, reported in Table 2. The highest values were registered for albumin extracts of both Finola and Secuieni J. seeds from the experimental field of Viganella. Comparing seeds of the same origin (certified seeds or seeds harvested from the field), albumin extracts of Secuieni J. variety were a bit more active than those of Finola, whereas no differences were observed in globulin extracts.

DPPH is a stable radical that can be reduced to a non-radical form in the presence of a hydrogen-donating antioxidant. The antioxidant activity of hempseed extracts determined using DPPH assay is indicated in Table 3. Albumin extracts obtained from seeds of the experimental field showed higher radical scavenging potential than certified seeds. The same trend was observed for globulin extracts of Finola seeds, while no differences were observed between extracts from certified and harvested seeds of Secuieni J.

### 2.3. SDS-PAGE Protein Analysis

**Figure 1:**
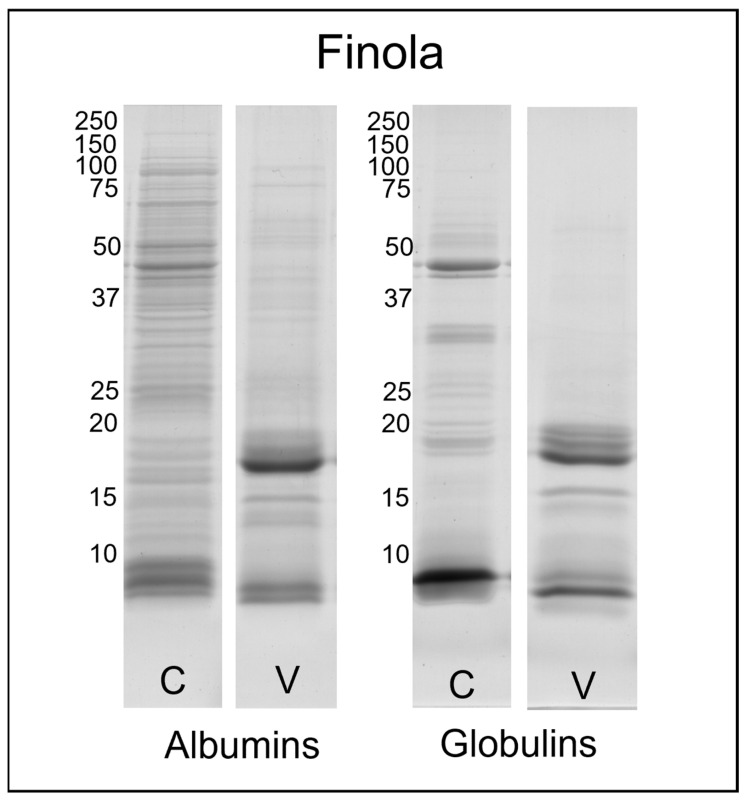
SDS-PAGE profile of albumin and globulin extracts obtained from Finola certified (C) and harvested seeds from the field of Viganella (V). The molecular weights (left) are expressed in kDa.

**Figure 2:**
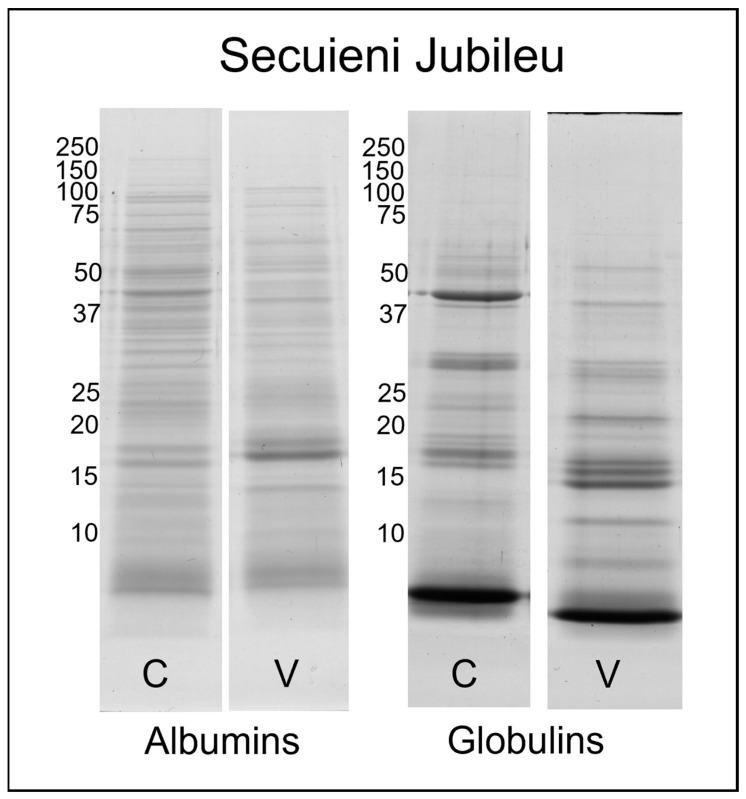
SDS-PAGE profile of albumin and globulin extracts obtained from Secuieni Jubileu certified (C) and harvested seeds from the field of Viganella (V). The molecular weights (left) are expressed in kDa.

Electrophoretic analyses were performed to determine the molecular weight distribution of proteins in the sample fractions. The SDS-PAGE profiles of the water-soluble (albumins) and salt-soluble (globulins) proteins from seeds of Finola and Secuieni J. varieties, of certified source (C) or from the experimental field (V), are presented in Figure 1 and Figure 2. The related uncropped gels are reported in Supplementary Figures S2 and S3.

Albumins show a higher number of bands than the fraction of globulins. Certified seeds of Finola and Secuieni J. varieties shared a similar protein profile obtained by SDS-PAGE. More than 30 bands were observed for the albumin fraction, with a molecular weight spanning from 150 to <10 kDa. The most intense bands were detected at 50, 48 and three main bands below 10 kDa, similarly to those reported for hempseed albumin 2S [18].

*C. sativa* albumin 2S (Cs2S) belongs to the plant lipid transfer protein (LTP) family, a ubiquitous multigene family of molecules expressed in different plant tissues with important roles in defense against biotic and abiotic stress, lipid binding and nutrient reservoir [19]. It is synthesized as a single polypeptide of 16 kDa and then subjected to proteolytic cleavage, for the removal of the N-terminal signal sequence and for splitting into small and large subunits, with a theoretical molecular weight of 3.77 and 7.67 kDa, respectively [20]. Cs2S shows the traits of a typical albumin 2S, with eight conserved cysteine residues forming two intra-chain disulfide bonds within the large subunit and two inter-chain disulfide bonds linking the small and large subunits in the mature protein. The molecular weight of the mature protein estimated by SDS-PAGE under non-reducing conditions analysis is about 10 kDa, while the two subunits migrate as bands of less than 6 kDa under reducing conditions [18,20]. Regarding the globulin fraction, 23–25 bands were detected, but the most intense were at 48, 35, 20, 18 kDa and one intense band below 10 kDa. As previously reported, the 48 kDa band corresponds to the vicilin C72-like protein, while 35 and 20–18 kDa bands are related to edestin, the main storage protein of hemp seeds [21,22]. In its native form, edestin is an hexamer composed of six subunits where each subunit (50 kDa) consists of an acidic chain (30–35 kDa) and a basic chain (18–20 kDa) connected via disulfide bonds [23]. The reducing conditions used for SDS-PAGE have dissociated these chains, which migrated in different positions of the gel.

Interestingly, the protein profile of seeds harvested from plants grown in the Viganella site differs from that of certified seeds, and this pattern is similar for both Finola and Secuieni J. seeds, but more pronounced in Finola extracts. In fact, both albumin and globulin fractions of harvested seeds showed fewer bands at high molecular weight, with the complete disappearance of bands previously detected at 48 and 35 kDa in Finola certified seeds. Meanwhile, more intense bands developed around 18–20 kDa and below 15 kDa with respect to certified seeds. These bands at lower molecular weight have been previously identified in Finola seeds as fragments of edestin [21]. Secuieni J. fractions from harvested seeds show a decrease in intensity of bands at 48 and 30–35 kDa, and a concurrent increase in those below 20 kDa.

### 2.4. MALDI Mass Spectra Analyses

**Figure 3:**
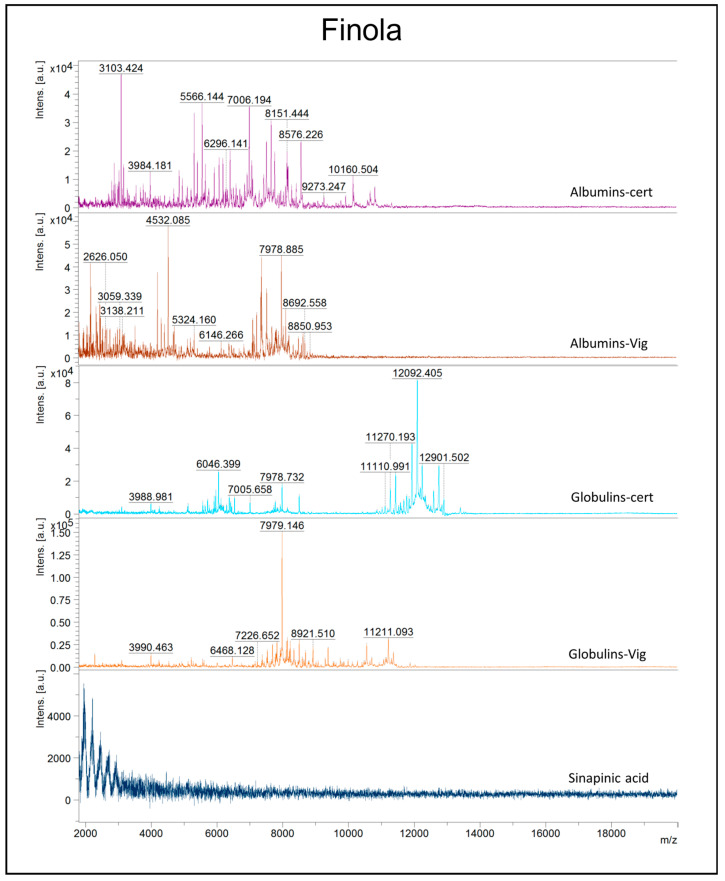
Representative MALDI-TOF MS mass spectra in the m/z region 2000–20,000 for protein extracts (albumins/globulins) of Finola seeds from commercial source (cert) or experimental field (Vig). The mass spectrum of the matrix (sinapinic acid) is also presented as reference.

**Figure 4:**
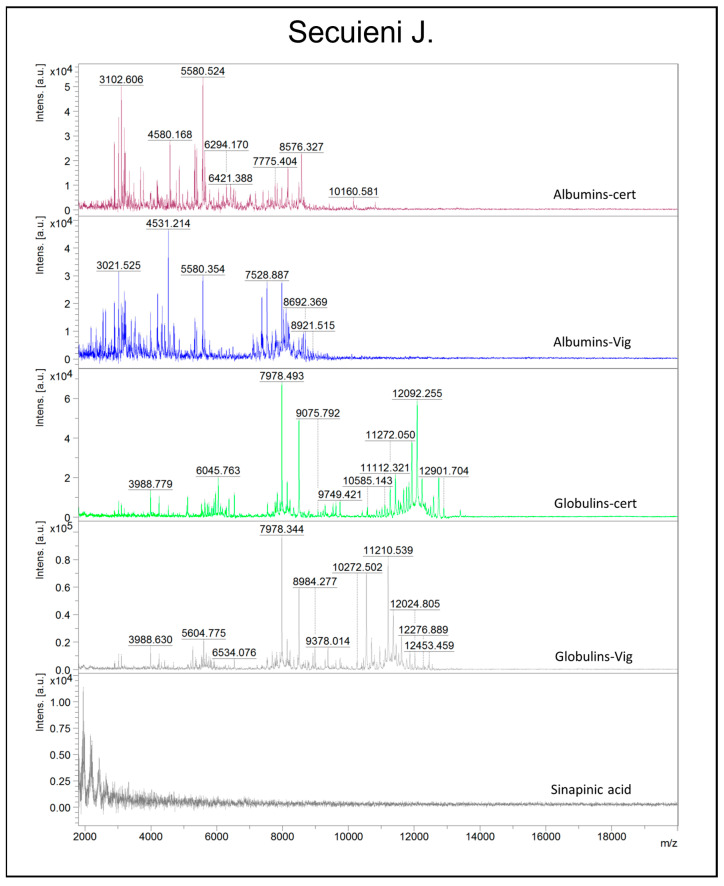
Representative MALDI-TOF MS spectra in the m/z region 2000–20,000 obtained for protein extracts (albumins/globulins) of Secuieni J. seeds from commercial source (cert) or experimental field (Vig). The mass spectrum of the matrix (sinapinic acid) is also presented as reference.

Polyacrylamide gel electrophoresis shows limited resolving power for low-molecular-weight molecules; thus, a more powerful technique, MALDI-TOF mass spectrometry (MS), was adopted. Albumin and globulin extracts from certified and harvested seeds of Finola and Secuieni J. varieties were obtained from independent extractions to assess the reproducibility of the extraction procedure. Nine spectra replicates were collected from each sample to confirm the reproducibility of the mass profile, and a total of 432 mass spectra were analyzed. One of the aims of the study was to assess the suitability of MALDI-TOF MS to discriminate seed protein extracts according to their variety and/or origin. To execute this, the mass range was chosen between 2 and 20 kDa since this mass range is not well resolved via SDS-PAGE; thus, more detailed information could be achieved by using MALDI-TOF MS as a complementary approach for protein analysis. Moreover, focusing on low masses could be useful to rapidly detect protein degradation events. Preliminary analysis of 1:1 sample/matrix mixtures resulted in spectra of poor quality (data not shown), while the dilution of albumin and globulin extracts with 10 μL of matrix solution was revealed as a quick and efficient strategy to obtain acceptable results. Albumins and globulins from certified seeds of Finola and Secuieni J. provided peculiar spectra which are represented in Figure 3 and Figure 4. Mass peaks higher than 13,000 m/z were not detected in the samples tested. Albumins show predominant signals at medium-low m/z, from 3000 up to 11000 m/z, while the presence of signals as high as 13,000 m/z was observed for globulins. Signals with the highest intensity in albumin extracts from certified seeds were observed at 3102 m/z (Finola) and 5580 m/z (Secuieni J.), while the signal at 4532 m/z was prevalent in harvested seeds of both Finola and Secuieni J.

The most intense signal for globulin extracts of certified seeds was detected at 12,092 m/z, while it was absent in seed extracts from the experimental field.

The signal at 7979 m/z was detected in albumin and globulin extracts, with higher intensity in the globulin fraction, and its intensity increased in seeds of both varieties from the experimental field. The signals 12,092–6045 and 7979–3990 observed for globulin extracts of both varieties represents MH^+^ and [M + 2H]^2+^ molecular species, respectively.

MALDI-TOF mass spectra were pre-processed as indicated in Section 4. Since no signals could be observed above 13,000 m/z, the spectra were trimmed from 0 to 15,000 m/z. Quality control pre- and post-trimming were also performed, revealing an acceptable low number of potential outlier spectra (less than 1.8%; that is, 2 out of 108) for the comparisons tested. Considering all the analyzed spectra, 3 out of 216 (1.4%) and 2 out of 216 (0.9%) potential outliers were detected for albumin and globulin extracts, respectively.

**Figure 5:**
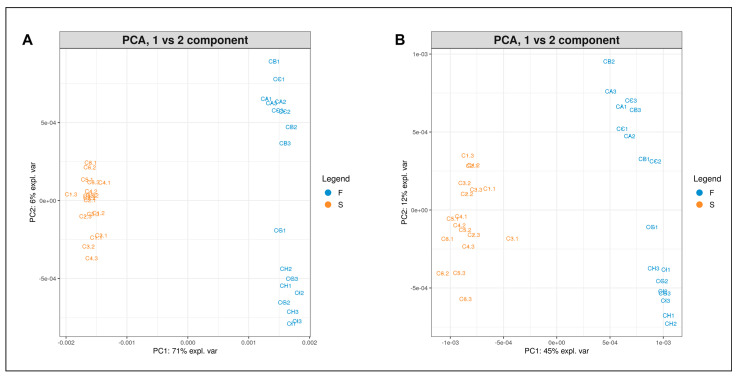
PCA plot (PC1 vs. PC2) of albumin (**A**) and globulin (**B**) extracts from certified seeds of Finola (F, blue) and Secuieni J. (S, orange) hemp varieties.

The analysis of the main components (PCA) was applied to the datasets of mass spectra of albumins and globulins from certified and harvested seeds of the two plant varieties, to detect sample differences or similarities related to the plant genotype or the growing site. A first comparison was made between Finola and Secuieni J. certified seeds to highlight the presence of signals that could be specifically related to the plant variety. Figure 5 shows the PCA plots (PC1 vs. PC2 component) of albumin (A) and globulin (B) protein profiles from certified seeds of Finola (F) versus Secuieni J. (S). The two groups were well distinguished, where PC1 and PC2 account for 76% and 67% of the total explained variance for albumin and globulin extracts, respectively.

The top loadings on the first and second principal components (PC1 and PC2) observed for albumin and globulin extracts of certified seeds are reported in Supplementary Figures S4 and S5, respectively. Among these, the top 25 loadings on the first principal component (PC1), representing the signals responsible for most of the variance observed, are reported in Table 4.

The distribution of the 25 most characteristic signals was almost equal in albumin extracts of both varieties (11 signals ascribed to Finola and 14 signals to Secuieni J.); the main difference between varieties was detected in globulin extracts: more signals were attributed to Secuieni J. than Finola seeds (19 vs. 6).

**Figure 6:**
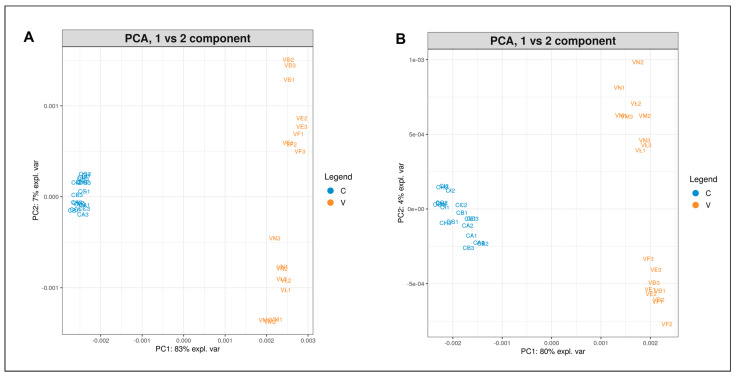
PCA plot (PC1 vs. PC2) of albumin (**A**) and globulin (**B**) extracts from Finola certified seeds (C, blue) and harvested seeds from the experimental field of Viganella (V, orange).

Considering one variety at a time, the albumin and globulin protein profiles of certified seeds were compared with those obtained from harvested seeds of the same variety to detect possible differences related to the growth environment. The PCA plots (PC1 vs. PC2 component) of albumin (A) and globulin (B) protein profiles from seeds of the Finola variety are depicted in Figure 6. Certified seeds and harvested seeds from the experimental field of Viganella (V) are clearly separated, with most of the variance explained by PC1. Viganella samples are more dispersed than the certified ones, with a total observed variance of 90% for albumins and 84% for globulin extracts.

**Figure 7:**
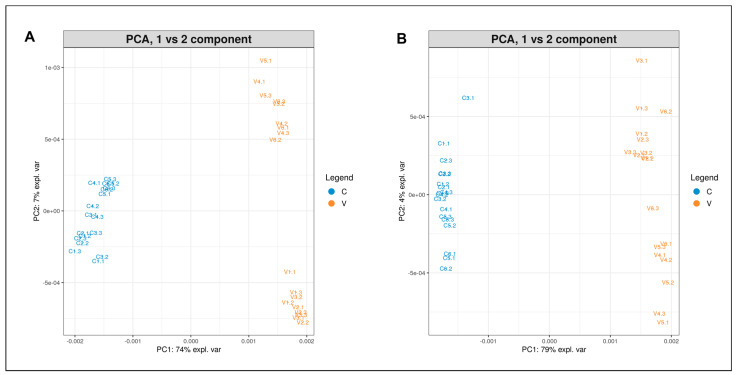
PCA plot (PC1 vs. PC2) of albumin (**A**) and globulin (**B**) extracts from Secuieni J. certified seeds (C, blue) and harvested seeds from the experimental field of Viganella (V, orange).

Regarding Secuieni J. variety, the PCA plots (PC1 vs. PC2 component) of albumin (A) and globulin (B) protein profiles are reported in Figure 7: certified seeds formed a distinct cluster compared to seeds from the experimental field (V), which are more dispersed than the certified ones, with a total variance of 81% for albumins and 83% for globulin extracts.

The top loadings on PC1 and PC2 observed for albumin and globulin extracts of Finola and Secuieni J. seeds are reported in Supplementary Figures S6–S9.

Table 5 reports the first 25 mass peaks responsible for most of the variance observed on the PC1 for albumin and globulin extracts of Finola and Secuieni J. seeds, highlighting their correlation with seed origin.

Considering seed origin, the main differences in the distribution of the 25 most characteristic signals were observed for albumin extracts, where a higher number of informative signals was ascribed to seeds from the experimental field compared to certified seeds (15 vs. 10 signals in Finola and 16 vs. 9 in Secuieni J. extracts). On the contrary, globulin extracts show a balanced distribution of peculiar signals between certified and harvested seeds of both varieties.

**Figure 8:**
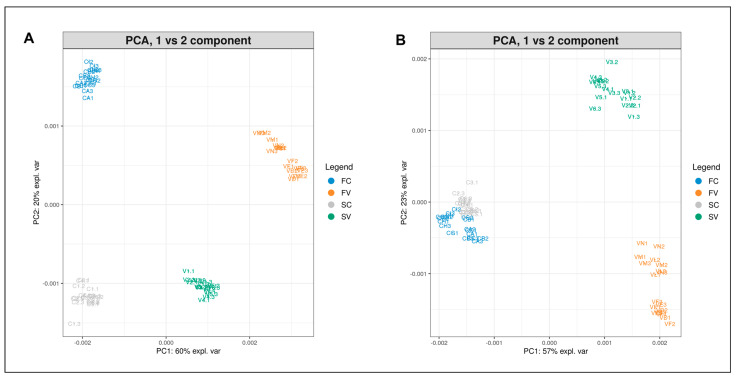
PCA plot (PC1 vs. PC2) of albumin (**A**) and globulin (**B**) extracts from Finola certified seeds (FC, blue), Finola harvested seeds from Viganella (FV, orange), Secuieni J. certified seeds (SC, gray) and Secuieni J. harvested seeds from the experimental field of Viganella (SV, green).

Finally, a comparison was made among spectra obtained from seeds of both varieties and origins. The PCA plots (PC1 vs. PC2 component) of albumin (A) and globulin (B) protein profiles from certified seeds (C) and harvested seeds from the experimental site (V) of Finola (F) and Secuieni J. (S) cultivars are reported in Figure 8. Four distinct groups can be observed, belonging to each variety and origin of seed, with a total variance of 80% for both albumins and globulins. The PC1 highlights differences related to seed origin (certified or harvested seeds), while the second principal component (PC2) shows variability traits related to the plant cultivar, which are more evident for albumins but not for the globulin extracts of certified seeds.

**Figure 9:**
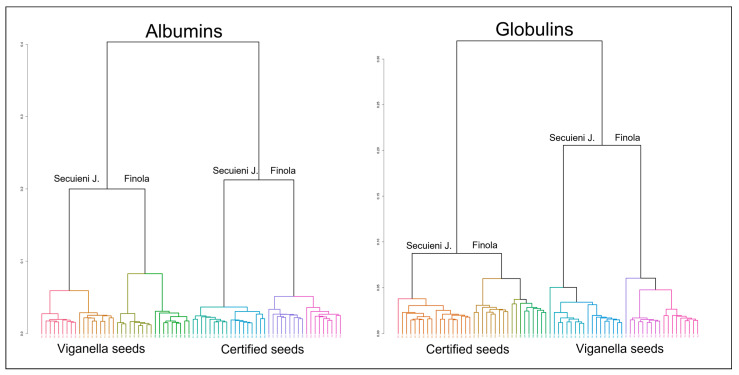
Dendrograms obtained by HCA of the mass spectra of albumin (left) and globulin (right) extracts from hemp seeds, according to their variety (Finola and Secuieni J.) and origin (certified and harvested seeds from the Viganella site).

Hierarchical clustering analysis (HCA) was performed to visualize the clustering of mass spectra. The two dendrograms generated using the data from seeds of different cultivar and origin are depicted in Figure 9. A clear distinction according to plant cultivar and seed origin is visible for both albumin and globulin extracts. The first ramification divides the samples obtained from the Viganella site and samples from certified seeds. Then, within each of these two groups, a second branching separates samples of Finola and Secuieni J. into two distinct clusters, formed by samples of the same type.

The analysis of the PC1 and PC2 top scores revealed differences with respect to plant cultivar and the growth environment; the most informative signals are indicated in Table 6.

Among the signals detected in certified seeds of both varieties, the ones at 3102, 2890 and 8577 m/z were observed in albumin extracts, while signals at 12,092, 6046, 6534, 12,241 and 5717 m/z were detected in globulin extracts, representing common traits of the analyzed cultivars’ seeds.

The signals shared among seeds of both Finola and Secuieni J. harvested from the experimental field include those at 4532, 7226, 7979, 7372, 7105 and 8594 m/z, observed in albumin extracts. Only one signal at 9379 m/z was detected in globulin extracts of both varieties.

## 3. Discussion

Hemp seeds represent a good source of nutrients due to their contents of highly digestible proteins and essential fatty acids. They also contain bioactive molecules, such as phenolic compounds and tocopherols, and also fatty acids and the products of protein digestion themselves.

It is well known that phenotypic traits are influenced by genotype, environment and their interactions. Hemp seed yield is strongly affected by climate parameters such as rainfall and temperature, with consequent variability in quality and quantity of the final product. In a study on five hemp cultivars under different growing environments, Ferfuia and collaborators [24] observed a considerable effect of environmental factors in determining variance in seed weight. One of the critical aspects was the temperature during the grain filling period, where an increase in mean temperature was related to a lower seed yield especially for medium-early varieties.

Beyond seed yield, environmental factors such as rainfall and temperature can affect the antioxidant activity, which is related to the profile of phenolic compounds. High rainfall during flowering and grain filling was found to increase the antioxidant capacity and phenolic content of hemp seeds [11].

Altitude is another factor that may exert an influence on the antioxidant capacity of plants, where low temperatures and high light intensity stimulate the production of antioxidant molecules. Rashid and collaborators [25] reported differences in the distribution and abundance of metabolites in *C. sativa* seeds from two geographical locations (subtropical and temperate climates). The exclusive or higher presence of metabolites such as phenols, flavonoids, vitamins and some amino acids (i.e., N, F, Y, S and homoserine) was detected in seeds from the high-altitude Himalayan temperate site compared with those from lower elevations. Altogether, these results suggest an important impact of the environment on the nutraceutical properties of seeds.

In the present study, a weight decrease was observed in seeds obtained from the experimental field compared with the certified ones, highlighting the effect of environmental conditions on both hemp varieties. To investigate possible differences in the antioxidant power, and with the aim of preserving the properties of proteins, hemp seed extracts were obtained using Osborne fractionation. This method was chosen since it works with mild conditions and allows the recovery of proteins in their native form, collecting different protein fractions according to their solubility.

Many studies carried out so far on hemp seed proteins have considered hemp protein isolates (HPI) as the starting material. HPI is obtained by applying acid-precipitation followed by protein extraction in alkaline solutions. Such extreme conditions cause alterations of the proteins’ structure, making it difficult to compare their properties with those of proteins in the native form that are contained in seeds consumed as a food. To our knowledge, only a few studies have analyzed the effects of different extraction methods on the final quality of hemp seed protein extracts, and the Osborne fractionation method resulted as an efficient alternative to acid precipitation and alkaline extraction [21,26,27,28].

Hemp seeds are rich in polyunsaturated fatty acids with high nutritional value but extremely prone to oxidation. The co-presence of antioxidant molecules such as γ- and α-tocopherol, polyphenols such as N-trans-caffeoyltyramine and cannabisin B, mainly found in the hull of hempseeds, and also *p*-hydroxybenzoic acid and catechin in the inner part of the seed contribute to prevent lipid degradation [29].

In this study, hemp seeds were defatted with hexane, thus removing the main part of those antioxidants associated with the lipid fraction such as γ-tocopherol.

The antioxidant power of water- and salt-soluble extracts of hemp seeds was determined with two widely used methods to assay the radical scavenging activity of plant extracts: DPPH and ABTS. In both cases, we observed higher reducing power values for albumin extracts obtained from seeds of the experimental field compared to certified seeds. These results could be due to the different presence of water-soluble antioxidants coming from the hull or internal parts of the seed. In fact, beyond the antioxidants present in hempseed oil, several bioactive compounds have been detected in hempseed cake, the by-product obtained after oil extraction. The principal phenolic compounds isolated from hemp seeds belong to the phenylpropionamide class, including phenylanamides and lignanamides. However, a study on phenolic antioxidant extraction reports that pure water extracts from defatted hemp seeds contain mainly phenolic acids such as *p*-coumaric and ferulic acid, while lignanamides and N-trans-caffeoyltyramine were not detected due to their scarce solubility in this solvent [30]. The absence of phenylpropionamides in water extracts confirms the poor capacity of water in extracting phenols, but is in contrast with the high total phenolic content estimated in water extracts with the Folin–Ciocalteu assay. The authors suggest that these results could be ascribed to the reducing behavior of other compounds such as sugars, organic acids and aromatic amines, which could be effectively extracted in water [30].

Proteins and peptides have demonstrated antioxidant activity as well: the DPPH radical scavenging activity of edestin and peptides obtained from hemp seed protein isolate has been documented. In general, it was observed that an increase in the radical scavenging activity goes along with protein hydrolysis, with higher activity related to smaller size peptides [31]. Hemp protein hydrolysates obtained with alcalase and flavourzyme showed higher DPPH scavenging activity than hemp protein isolates, suggesting that the hydrolysis process releases peptides that can react with free radicals, enhancing the overall antioxidant activity [32]. Hemp seed bioprocessing through fermentation is another effective way to increase the presence of bioactive compounds, as a result of the metabolic actions of microorganisms aimed at recovering nutrients from the substrate or at detoxifying molecules. Peptides belonging to edestin isoforms (AQVSVGGGR and DLQIIAPSR) have been identified from fermented hemp fractions showing high DPPH radical scavenging activity. These peptides are characterized by high contents of hydrophobic and branched-chain amino acids, which could explain their antioxidant activity [33]. Interestingly, the same peptides have been identified in the low-molecular-weight protein fraction of Finola seeds obtained from the same experimental field of this study after 2D-gel protein analysis, as previously reported in [21]. Therefore, the differences here observed for the ABTS and DPPH antioxidant power of extracts from certified and harvested seeds, and especially the higher DPPH activity of Finola versus Secuieni J. extracts, could be also related to an increased presence of such peptides in seeds of the mountain site.

The SDS-PAGE protein profiles of albumins and globulins of certified seeds are in line with those reported in the literature for hemp seeds [22,27], with albumin extracts showing the highest number of bands. The use of conventional buffers for protein extraction (e.g., SDS-PAGE lysis buffer) could result in the under-representation of proteins from the albumin family in the low molecular weight range, with loss of information regarding species-specific proteins, as pointed out by a proteomic analysis of oilseed cake from different species [34]. By applying the Osborne method, it was possible to obtain extracts rich in active molecules and in otherwise low-abundant proteins.

Under reducing conditions, bands related to vicilin C72-like protein (48 kDa) and albumin 2S (<10 kDa) were detected in water-soluble extracts, while salt-soluble extracts were mainly represented by bands related to vicilin C72-like and edestin (30–35 and 18–20 kDa). A decrease in high-molecular-weight bands with a concomitant increase in low-molecular-weight bands was observed in harvested seeds from the experimental field; however, it was not possible to discriminate signals below 10 kDa by the use of this technique.

The MALDI-TOF approach allowed a more detailed evaluation of the low-molecular-weight fraction of the samples, highlighting different peak patterns and the increase in signals at low m/z in seed extracts from the experimental field, with the lowest m/z values observed in the albumin fraction. PCA analysis clearly distinguished seed extracts based on their origin (certified or experimental field) and cultivar, with the highest discriminating power observed for albumin extracts of Finola seeds. This result could be in part due to the higher variety of proteins contained in the water-soluble fraction compared to the salt-soluble globulins. HCA analysis gave back a clear separation of samples according to their origin and plant cultivar, suggesting the adequate performance of the adopted method in discriminating similar but not identical samples. The analysis of mass spectra allowed the detection of conserved mass peaks in Finola and Secuieni J. albumin (3102, 2890, 8577 m/z) and globulin extracts (12,092, 6046, 6534, 12,241, 5717 m/z), which could provide useful information to detect hemp seed proteins in foods. Moreover, other signals more specifically related to hemp cultivar or seed source were detected.

Structural properties such as amino acid composition and length are features of recognized importance in determining the nutritional and bioactive role of proteins. Edestin, the main storage protein of hempseed, is rich in arginine and presents a higher arginine/lysine ratio than soybean proteins, with a role in the maintenance of normal blood pressure and having potential positive effects on the cardiovascular system. The isoform 3 of edestin and Cs2S are sulfur-rich proteins, with sufficient methionine content and therefore enhanced nutritional value. Moreover, differences in the amino acid sequence of proteins belonging to the same family, such as Cs2S or Cs7S vicilin-like protein, have been observed in seeds of different hemp varieties from distant geographical regions, highlighting the role played by plant genotype and growth area in determining protein and peptide bioactivity [27].

Antioxidant and antihypertensive activities of peptides are related to the presence of hydrophobic, acidic, aromatic and branched-chain amino acids [9]. A possible explanation for the higher radical scavenging activity observed here for albumin extracts compared to globulins lies in the amino acid composition of these proteins. Cs2S, the most prevalent protein in water extracts, presents the highest level of sulfur-containing amino acids when compared to Cs7S and Cs11S globulins, suggesting a better antioxidant effect, as reported in [35]. The sequence of amino acids is another significant trait in determining potential bioactivity: for instance, hydrophobic amino acids at the N-terminus and hydrophilic amino acids at the C-terminus of a peptide have been correlated with high free radical scavenging activity [36]. The abundance of low-molecular-weight compounds revealed by mass analysis in the albumin fraction, together with the previous identification of edestin peptides with antioxidant activity in the protein extracts from the experimental field [21], further support the hypothesis that the higher radical scavenging activity of albumin extracts is at least in part due to molecules of protein origin.

Moreover, MALDI-TOF analysis of albumin and globulin extracts could be an efficient and rapid way to highlight minor changes in the protein profile of hemp seeds, identifying signals which could be related to protein isoforms and possibly used as markers of hemp. The promising results of this approach could be further applied to an increased number of hemp cultivars.

## 4. Materials and Methods

### 4.1. Plant Material and Experimental Field

Two *C. sativa* cultivars were chosen for this study: the dioecious Finola and the monoecious Secuieni Jubileu. Seeds of the Finola hemp variety were kindly provided by ArsUniVCO, an association for culture development of university studies and research in the Verbano Cusio Ossola area (Italy), while seeds of the Secuieni Jubileu hemp variety were purchased from a commercial company (Canapuglia, Bari, Italy). The experimental field is located in the municipality of Viganella, a small village (hamlet of Borgomezzavalle) surrounded by the mountains in the middle of Valle Antrona, Italy (latitude 46°03′09.3″ N, longitude 8°11′37.0″ E, elevation 583 m a.s.l.), within Alpi Pennine and Lepontine, in the Northwestern Alps subsection, and belonging to the temperate semi-continental bioclimate [37]. *C. sativa* plants were grown in 5 m × 5 m plots obtained from the terraced mountainside, without fertilization or irrigation during the growing season. The sowing took place in the last two weeks of May 2018 (Finola) and 2019 (Secuieni J.). The two varieties were sown with a pattern of 20 cm between rows and at intervals of 15 cm within each row. The seeds were manually harvested at maturity and left to dry in a cool, dry room for a couple of weeks. Measurements of the temperature (°C) and rainfall (mm) were provided by a meteorological sensor from a regional network (ARPA Piemonte, Agenzia Regionale per la Protezione Ambientale). The meteorological sensor nearest to Viganella is “Alpe Cheggio”, in the municipality of Antrona Schieranco (latitude 46°05′06″ N, longitude 08°06′56″ E, 1460 m a.s.l.).

### 4.2. Seed Weight

The weight of seeds used for the sowing (henceforth denoted as “certified seeds”) and seeds obtained from the experimental field (denoted as “harvested seeds”) was assessed from 50 randomly selected seeds, weighed in triplicate using an analytical balance (Quintix, Sartorius, Varedo, Italy).

### 4.3. Protein Extraction

Protein fractions were obtained from certified and harvested seeds of Finola and Secuieni Jubileu cultivars following the Osborne fractionation method [38] with certified seeds used as a reference control. Water-soluble (albumins) and salt-soluble (globulins) protein fractions were collected for the aim of this study. Two independent protein extractions, with three replicates each, were performed as described in [28]. Briefly, one gram of hemp seeds was ground in a mortar at 4 °C. Hempseed powder was defatted with hexane by shaking overnight at 250 rpm (PSU-20i, Biosan, Riga, Latvia). Hexane was decanted and the seed pellet was left to dry to remove the remaining solvent. The albumin fraction was recovered by adding 1 mL of ultrapure to 0.1 g of hempseed powder and vortexed for 5 min (ZX3, Advanced Vortex Mixer, VELP SCIENTIFICA, Usmate, Italy), then the tubes were left on ice on a shaker (PSU-20i, Biosan, Riga, Latvia) for 55 min at 250 rpm. The tubes were centrifuged at 12,000× *g*/4 °C for 10 min (Fresco 21, Thermo Fisher Scientific, Waltham, MA, USA) and the supernatant was transferred into new tubes and stored at 4 °C. A second extraction of the pellet was performed by adding 1 mL of water and repeating the procedure described above. The second supernatant was added to the previous one: an aliquot was kept at 4 °C and analyzed within 24 h for mass spectrometry and antioxidant activity assays, and the remainder was stored at −20 °C until further use. The pellet was washed with 1 mL of ultrapure water, vortexing the tubes for 5 min and centrifuging at 12,000× *g*/4 °C for 10 min. The supernatant was discarded and the globulin fraction was extracted by adding 1 mL of NaCl 5% to the pellet and vortexing for 5 min. Then, the tubes were left on a shaker for 55 min at 250 rpm and finally centrifuged at 12,000× *g*/4 °C for 10 min. The supernatant was finally transferred into new tubes and stored as indicated for the albumin fraction. The protein content of different extracts was estimated using the Bradford assay, with bovine serum albumin (BSA) as the protein standard [39].

### 4.4. Determination of Radical Scavenging Activity by ABTS and DPPH Assays

The antioxidant activity (AA) of water-soluble and salt-soluble fractions was determined by the ABTS (2,2-azinobis-[3-ethylbenzothiazoline-6-sulphonic acid) and DPPH (Diphenyl Picryl hydrazine) radical scavenging assays, using two methods adapted for 96-well microplates. The ABTS radical scavenging activity was determined according to [40], with some modifications. ABTS was dissolved in water to a final concentration of 7 mM. The ABTS solution was mixed 1:1 with potassium persulfate (4.9 mM) and left in the dark at room temperature for 16 h. The reaction of ABTS with potassium persulfate produced the ABTS radical cation (ABTS**⸱**^+^) and this solution was filtered and diluted to an absorbance of 0.70 at 734 nm. Then, 190 μL of diluted ABTS**⸱**^+^ working solution was mixed with 20 μL of properly diluted sample solution. The final solution was incubated in the dark at room temperature for 7 min and then transferred into a 96-well microplate. The absorbance was read at 734 nm with a microplate reader (Spark 10 M, Tecan, Mannedorf, Switzerland), repeating the measure three times for each sample replicate.

The radical scavenging activity was calculated as the percentage reduction in ABTS**⸱**^+^ according to the formula:

(1) $$\%\;\mathrm{Reduction}=\frac{\mathrm{Absorbance}\;\mathrm{of}\;\mathrm{blank}\;-\;\mathrm{Absorbance}\;\mathrm{of}\;\mathrm{sample}}{\mathrm{Absorbance}\;\mathrm{of}\;\mathrm{blank}}\times 100$$

The DPPH antioxidant activity was determined according to [41,42] with some modifications: DPPH was dissolved in methanol to a final concentration of 0.625 mM. The stock solution containing DPPH radicals was diluted to an absorbance of 1.0 at 517 nm. Then, 190 μL of diluted DPPH working solution was mixed with 20 μL of properly diluted sample solution and left in the dark at room temperature for 16 h. The final solution was transferred into a 96-well microplate and the absorbance was read at 517 nm by using a microplate reader. The measurement was repeated three times for each sample replicate.

The radical scavenging activity was calculated as the percentage reduction in DPPH⸱according to the formula:

(2) $$\%\;\mathrm{Reduction}=\frac{\mathrm{Absorbance}\;\mathrm{of}\;\mathrm{blank}\;-\;\mathrm{Absorbance}\;\mathrm{of}\;\mathrm{sample}}{\mathrm{Absorbance}\;\mathrm{of}\;\mathrm{blank}}\times 100$$

Trolox was used as a reference compound and the results of the ABTS and DPPH assays are expressed as μM of Trolox equivalents (TE)/mg of sample.

### 4.5. Protein Electrophoresis

Hempseed proteins resulting from sequential extraction were analyzed by sodium dodecyl sulphate–polyacrylamide gel electrophoresis (SDS-PAGE). Ten micrograms of each protein extract was mixed with Laemmli buffer (2% *w*/*v* SDS, 10% glycerol, 5% 2-mercaptoethanol, 62 mM Tris-HCl pH 6.8) and loaded onto 10 × 8 cm vertical 12% polyacrylamide gels. Protein standards (Precision Plus Protein Dual Color, Biorad, Hercules, CA, USA) were used to estimate the molecular weight of proteins. The electrophoretic run was performed at 120 V for 2 h with a Mini Protean System (BioRad, Hercules, CA, USA). Gel staining was performed with Colloidal Coomassie brilliant blue G-250 and the gel image was acquired by a GS-900 densitometer using the software ImageLab (BioRad, Hercules, CA, USA).

### 4.6. Sample Preparation for MS Analysis

The sample preparation was optimized in terms of sample dilution, matrix of choice and solvent used. Albumin and globulin fractions (1.5 μL) obtained from each sample were mixed with 10 μL of matrix solution (10 mg/mL sinapinic acid dissolved in acetonitrile: 0.1% TFA, 30:70% *v*/*v* -TA30) and 1 μL of this mixture was spotted on a ground steel MTP 384 target plate (Bruker Daltonics GmbH, Bremen, Germany). All sample replicates were spotted nine times to ensure the repeatability of the method. The target plate was allowed to dry at room temperature before MALDI-TOF MS analysis.

### 4.7. MALDI-TOF MS Analysis

MALDI-TOF mass spectra of protein extracts were acquired using an Ultraflextreme mass spectrometer (Bruker Daltonics GmbH, Bremen, Germany) equipped with a Smartbeam 2 Nd:YAG laser operating at 355 nm, using FlexControl software (version 3.3, Bruker Daltonics GmbH, Bremen, Germany). The instrument was operated in linear mode with positive ion acquisition in the 2000−20,000 m/z range, using the following parameters: laser attenuator range, 40% to 80%; laser power: 80%, laser frequency: 2000 Hz; processing method parameters: s/*n* = 3, relative intensity threshold = 0%, minimum intensity threshold = 0, maximum number of peaks = 100, peak width = 5 m/z, height = 80%. The instrument voltages were as follows: ion source 1: 20.04 kV; ion source 2: 18.84 kV; lens: 5.67 kV. Pulsed ion extraction: 250 ns. Deflection of matrix ions up to 900 Da was enabled to avoid detector saturation. Mass spectra were collected summing 6000 shots per sample; random walk with partial sample mode was enabled. Finally, 54 spectra (6 independent sample extracts, 9 technical replicates each) were collected from each protein fraction (albumins/globulins) of Finola and Secuieni J. certified and harvested seeds. Mass spectra were calibrated using “Protein Calibration Standard I” (Bruker Daltonics GmbH, Bremen, Germany) in FlexAnalysis software (version 3.3, Bruker Daltonics GmbH, Bremen, Germany).

### 4.8. MS Data Processing

Information related to mass spectra (m/z and intensity) was exported and analyzed using the online platform GeenaR [43]: a target file, that is, a tab-separated value file containing the spectra and sample names, the number of replicates and the sample group, was created and loaded on the platform, together with the dataset folder containing the sample spectra in the form of text files. The following parameters were applied for the pre-processing of spectra: trimming from 0 to 15,000 m/z; variance stabilization: square root (sqrt); smoothing: Savitzky–Golay with half window size of 10; baseline removal: TopHat, with half window size of 75; normalization: total ion current (TIC). An average mass spectrum for each sample was obtained by calculating the mean of intensity for each m/z value of the technical replicates. Peak alignment was performed using MAD (median absolute deviation) for noise estimation (half window size: 20, SNR: 2) and LOWESS (local weight scatterplot smoothing) for phase correction (mass tolerance: 0.002). Then, peaks were selected by applying the strict method for binning and a coverage (minimum frequency of peaks for their selection) of 0.5. A feature (peak) matrix was generated, and a heat map was also provided to see the peak distribution. Principal component analysis was applied on the pre-processed mass spectra. Hierarchical clustering analysis was performed using the average linkage method and the gap statistic to estimate the number of clusters. The dendrogram was created by using the cosine correlation as a similarity measure between two samples (peak list).

Data are expressed as mean ± standard deviation of the mean. Statistical analysis of data was performed by using one-way analysis of variance (ANOVA) and Tukey’s test, with *p*-values < 0.05 considered statistically significant.

## 5. Conclusions

In this work, the Osborne method was effective in obtaining bioactive extracts enriched in low-abundant proteins. Albumin extracts from seeds of the mountain site showed higher reducing power compared to certified seeds, while only minor differences were observed among the two cultivars. These results could be explained by a combination of factors, including different amounts of water-soluble phenols and the presence of peptides belonging to edestin isoforms. The MALDI-TOF approach highlighted the presence of peculiar peak patterns among seed extracts, and demonstrated acceptable performance in discriminating samples according to their origin and plant cultivar. Conserved mass peaks were detected in seeds of Finola and Secuieni Jubileu, with possible use as markers of hemp seed proteins in foods. Moreover, the highest variance observed among mass spectra was related to seed origin rather than plant cultivar, confirming a strong effect played by the growth environment on the qualitative and nutraceutical traits of hemp seeds.

## Figures and Tables

**Table 1:** Mean weight (g) and standard deviation (SD) of 50 seeds from Finola and Secuieni J. varieties, obtained from the experimental field of Viganella, and certified seeds included as a reference. The decrease in weight of harvested seeds compared to certified seeds is shown as a percentage reduction.

Variety	Source	Mean ± SD	% Difference vs. Certified Seeds
Finola	Certified	0.62 ± 0.02 a	
	Viganella	0.47 ± 0.05 b	−23
Secuieni J.	Certified	0.88 ± 0.01 c	
	Viganella	0.48 ± 0.01 b	−44

Statistical differences after ANOVA (*p* < 0.05) are expressed by different letters. These values are a mean of three sample replicates ± SD (standard deviation).

**Table 2:** Antioxidant properties of albumin and globulin hempseed extracts determined by ABTS assay and expressed as μMTE/mg sample.

ABTS μMTE/mg	Albumins		Globulins	
	Secuieni J.	Finola	Secuieni J.	Finola
Certified	6.31 ± 0.40 aA	5.44 ± 0.49 bA	3.23 ± 0.06 cA	3.12 ± 0.16 cA
Viganella	8.50 ± 0.41 aB	7.50 ± 0.54 bB	2.72 ± 0.16 cB	2.93 ± 0.22 cA

Statistical differences after ANOVA (*p* < 0.05) are expressed by different letters: horizontal comparisons are represented by lowercase letters while capital letters indicate vertical comparisons. These values are a mean of six sample replicates ± SD (standard deviation).

**Table 3:** Antioxidant properties of albumin and globulin hempseed extracts determined by DPPH assay and expressed as μMTE/mg sample.

DPPH μMTE/mg	Albumins		Globulins	
	Secuieni J.	Finola	Secuieni J.	Finola
Certified	2.63 ± 0.42 aA	2.58 ± 0.26 aA	0.69 ± 0.1 bA	0.75 ± 0.12 bA
Viganella	5.21 ± 0.28 aB	8.16 ± 0.73 bB	0.65 ± 0.13 cA	1.09 ± 0.06 dB

Statistical differences after ANOVA (*p* < 0.05) are expressed by different letters: horizontal comparisons are represented by lowercase letters while capital letters indicate vertical comparisons. These values are a mean of six sample replicates ± SD (standard deviation).

**Table 4:** Mass signals from albumin and globulin extracts of certified seeds corresponding to the top 25 loadings on PC1 and their correlation with plant variety.

Protein Extract	Finola	Secuieni J.
	3039.2727	5580.8948
	7665.5076	5339.4866
	5566.0499	3194.9156
	6084.6582	3021.9314
	5324.4096	4580.8411
	7522.8941	5636.0606
Albumins	2925.5656	5394.9022
	5937.8299	8496.3288
	8183.0591	7838.2702
	7006.6362	3483.4856
	6199.3435	7978.9395
		4487.9038
		3223.5109
		5619.329
	5565.3173	8500.5256
	12842.0316	5537.9171
	7006.5751	7979.0936
	6375.353	6534.1247
	3177.0887	8142.0715
	2988.1917	9750.0606
		3103.4136
		9534.8318
Globulins		7545.1828
		3022.3737
		9292.1121
		5089.1376
		4250.1509
		5119.1642
		9076.8148
		9621.3895
		8227.3841
		11182.0469
		8593.9064

**Table 5:** Mass signals from albumin and globulin extracts of Finola and Secuieni J. seeds corresponding to the top 25 loadings on PC1 and their correlation with seed origin (certified vs. Viganella).

	Finola		Secuieni J.	
Protein Extract	Certified	Viganella	Certified	Viganella
		4531.9135	4580.5108	7528.93
		7978.9816	3102.7574	7225.7856
	3102.9927	7225.1839	3021.6484	7105.5253
	5566.1042	2164.6037	8577.3498	7371.5982
	5324.5555	7371.6336	3194.7097	8692.8527
	2890.8888	7105.0594	3348.128	4345.2583
	3176.8442	4206.6004	2890.547	4531.7882
Albumins	3039.4608	8593.0149	4865.0068	7978.7868
	6201.603	2182.0704	3773.8866	8108.0976
	3022.262	8105.0117		3987.8706
	8577.0498	6145.2977		8032.9275
	7664.2159	7352.2081		8593.7884
		2451.1964		2547.2683
		2469.5645		4693.4452
		2760.3171		8852.011
				2625.3849
	12091.5994	7978.9396	12092.7762	11210.5162
	6046.3194	8693.2093	11433.9663	10550.9063
	11928.9451	7821.9015	6046.1578	5605.2708
	11427.6743	8227.6458	11933.8185	5275.6731
	5914.7622	9379.1567	11273.8292	8985.4836
	5964.3426	7686.0268	11843.027	11616.807
Globulins	12753.1011	7226.3202	12241.5303	11366.8126
	6533.8252	2279.7868	5716.8037	11870.0922
	11270.9	8921.4531	6533.9855	10706.9665
	12240.5771	8593.7362	11182.726	10464.123
	12591.7529	7373.7156	10586.1044	10956.6811
	12901.7371	7530.4124	12748.5806	9378.6813
	5716.7694			5683.5626

**Table 6:** Mass signals from albumin and globulin extracts corresponding to the top 25 loadings on PC1 and PC2 indicating their correlation with seed origin (certified vs. Viganella) and variety (Finola and Secuieni J.), respectively.

	PC1		PC2	
Protein Extract	Certified	Viganella	Finola	Secuieni J.
	3102.8323	4531.8509	5324.3517	5581.1447
	3021.8527	7225.9687	3039.197	5339.4398
	2890.6526	7978.8497	5565.9502	3194.9059
	5565.9502	7371.6373	6084.3232	3021.8527
	8576.9773	7105.2896	5425.0599	5394.8266
	4580.6829	2164.3503	7663.6769	5636.3499
	3194.9059	8108.0994	5937.361	4580.6829
Albumins	3348.1186	7355.598	6199.0767	3223.6196
	3177.1276	8593.9458		7978.8497
		8693.0508		3483.6592
		7529.3259		5619.0109
		2181.9145		4345.5345
		6145.9385		4189.3057
		4206.3774		7839.7865
		2451.4945		2907.6453
		2469.167		8165.0733
				7572.6529
	12092.5123	11210.8371	7822.1068	11210.8371
	11433.226	10550.6548	7978.9754	10550.6548
	6046.3123	8693.1088	8693.1088	5537.7865
	11932.6533	7978.9754	8108.3643	3022.2238
	6534.0306	9378.618	7530.5073	5605.3792
	11272.522	7822.1068	2279.2525	5275.621
	12241.1361	8227.7062	8593.8747	5762.6403
	5966.3936	7686.0982	7226.2781	11617.3375
Globulins	5716.9575	7226.2781		8985.339
	5915.2323	8921.9322		5915.2323
	11842.4393	2279.2525		11366.8715
	12902.2419	5605.3792		10707.0087
	12591.3015			10463.8831
				6534.0306
				8500.3492
				11870.7253
				5683.6641

## Data Availability

The data can be made available upon reasonable request.

## Supplementary Materials

The following supporting information can be downloaded at: https://www.mdpi.com/article/10.3390/plants12040782/s1, Table S1: Average air temperature and rainfall of the years 2018 and 2019, Viganella, Italy; Figure S1: Box plot representing the weight of 50 seeds of Finola and Secuieni J. hemp varieties. Harvested seeds from the experimental field (Viganella) are compared with certified (Cert) seeds as a reference; Figure S2: Uncropped gels obtained after SDS-PAGE of albumin and globulin extracts from Finola certified (C) and harvested seeds from the field of Viganella (V). Three replicates for each sample extract were run on the same gel; Figure S3: Uncropped gels obtained after SDS-PAGE of albumin and globulin extracts from Secuieni Jubileu certified (C) and harvested seeds from the field of Viganella (V). Three replicates for each sample extract were run on the same gel; Figure S4: Top loadings on PC1 and PC2 from PCA analysis of albumin extracts from certified seeds of Finola and Secuieni J. varieties; Figure S5: Top loadings on PC1 and PC2 from PCA analysis of globulin extracts from certified seeds of Finola and Secuieni J. varieties; Figure S6: Top loadings on PC1 and PC2 from PCA analysis of albumin extracts from certified and Viganella seeds of Finola variety; Figure S7: Top loadings on PC1 and PC2 from PCA analysis of globulin extracts from certified and Viganella seeds of Finola variety; Figure S8: Top loadings on PC1 and PC2 from PCA analysis of albumin extracts from certified and Viganella seeds of Secuieni J. variety; Figure S9: Top loadings on PC1 and PC2 from PCA analysis of globulin extracts from certified and Viganella seeds of Secuieni J. variety.
