# Supplementary material for: Protein Mass Fingerprinting and Antioxidant Power of Hemp Seeds in Relation to Plant Cultivar and Environment
Source: Plants (Basel). 2023 Feb 9;12(4):782. doi: 10.3390/plants12040782 (PMC9966504; doi:10.3390/plants12040782)
Supplement: Supplementary file 1 [file plants-12-00782-s001.zip › plants-2197375-supplementary.pdf]

Table S1. Averageair temperatureand rainfallof the years2018 and 2019, Viganella, Italy.

	Rainfall (mm)		Min Temperature (°C)		Max Temperature (°C)	
	2018	2019	2018	2019	2018	2019
April	-	-	-	-2.6	-	14.4
May	306.6	86.8	1	-1.2	19.8	17.1
June	97.8	192.8	7.1	-	25.5	-
July	94.2	134.4	11.2	7	26.6	25.4
August	155.6	137.4	7.5	8.8	26.5	22.3
September	60.8	68.4	4.6	5.4	21.4	21.5
Mean			6.28	3.48	23.96	20.14
Total	715	619.8				

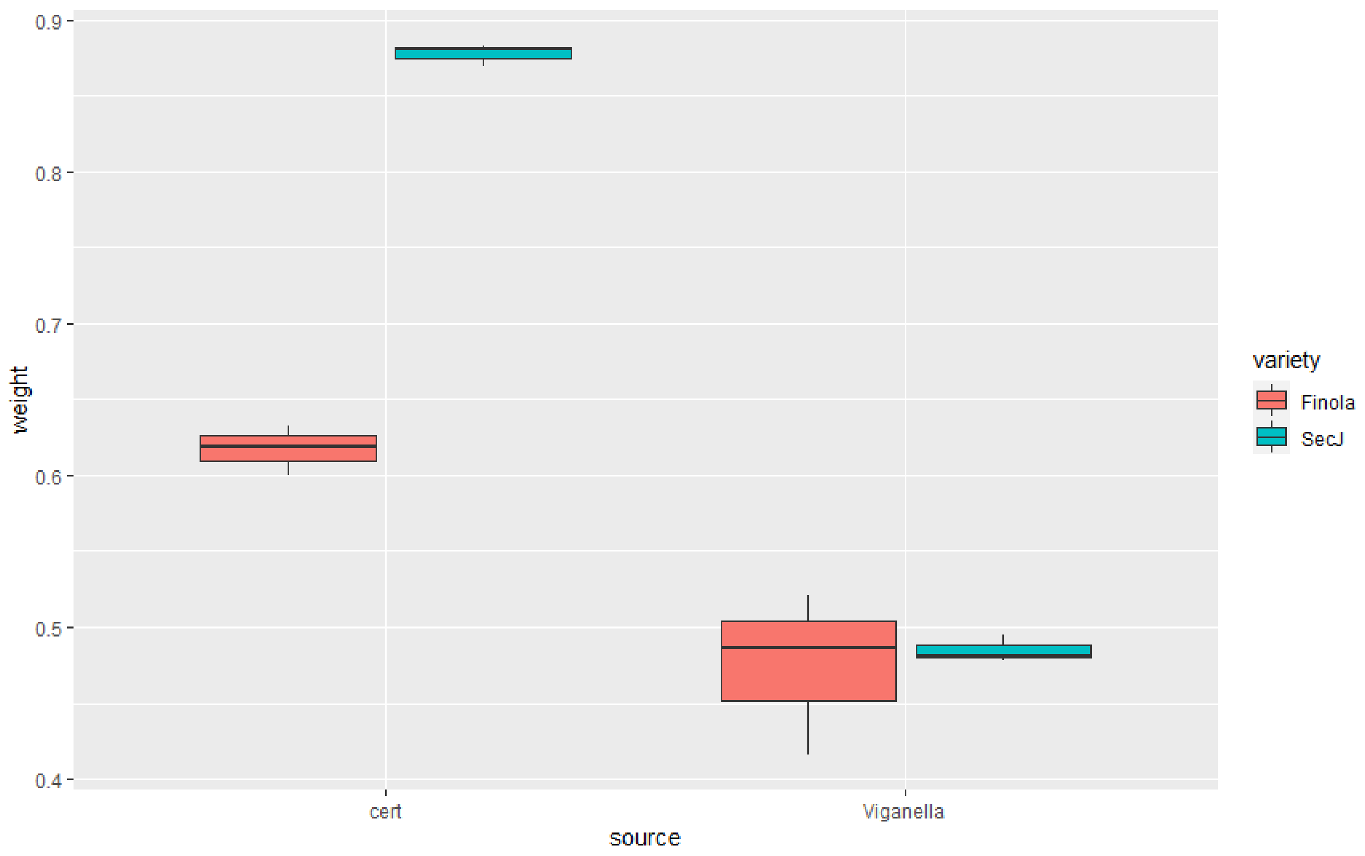

Figure S1. Box-plot representing the weight of 50 seeds of Finola and Secuieni J. hemp varieties. Harvested seeds from the experimental field (Viganella) are compared with certified (Cert) seeds as a reference.

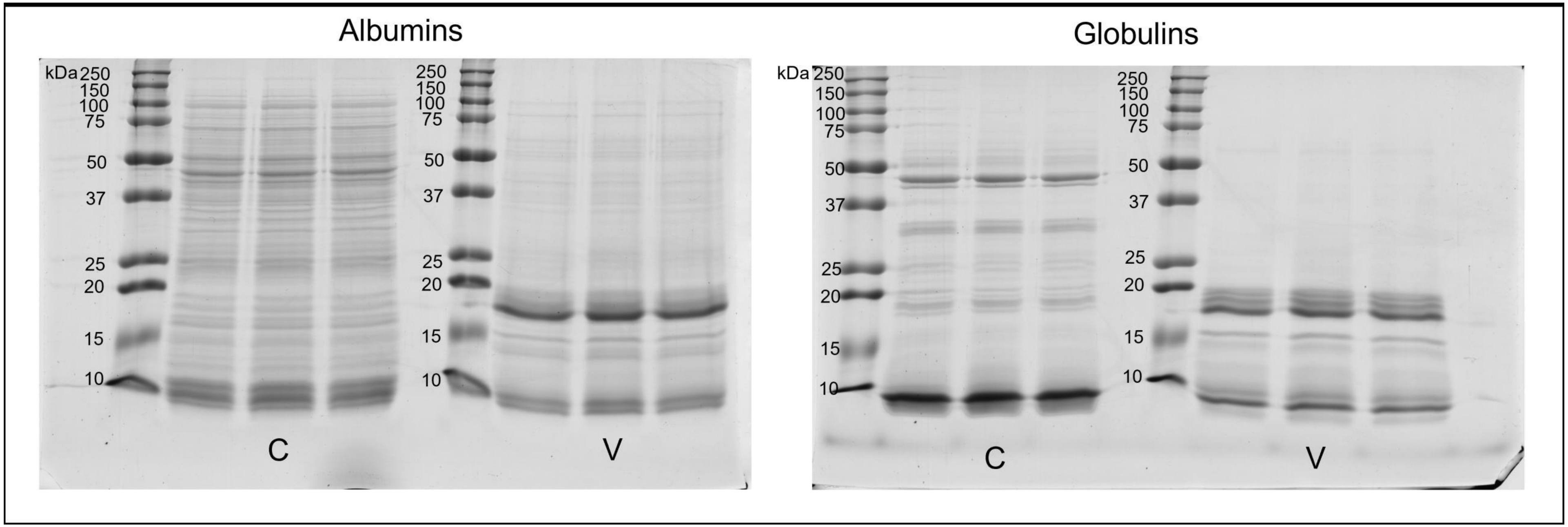

Figure S2. Uncroppedgelsobtainedafter SDS-PAGE of albumin and globulin extracts from Finola certified (C) and harvested seeds from the field of Viganella(V). Three replicatesfor eachsample extractwererunon the samegel.

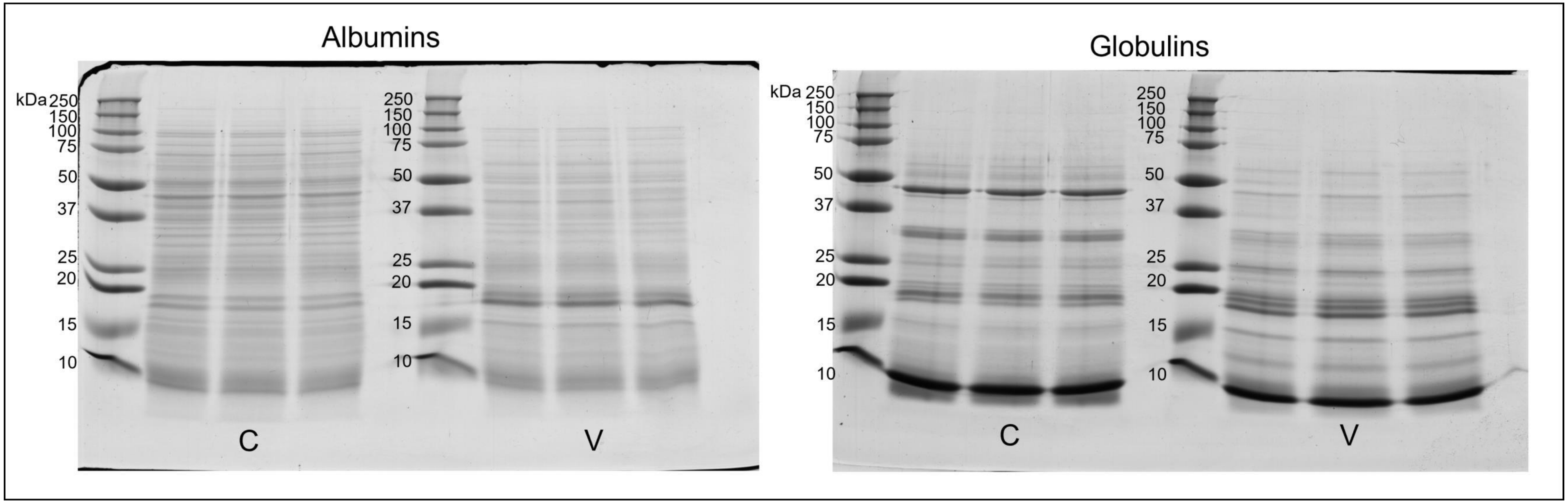

Figure S3. Uncroppedgelsobtainedafter SDS-PAGE of albumin and globulin extracts from SecuieniJubileucertified (C) and harvested seeds from the field of Viganella(V). Three replicatesfor eachsample extractwererunon the samegel.

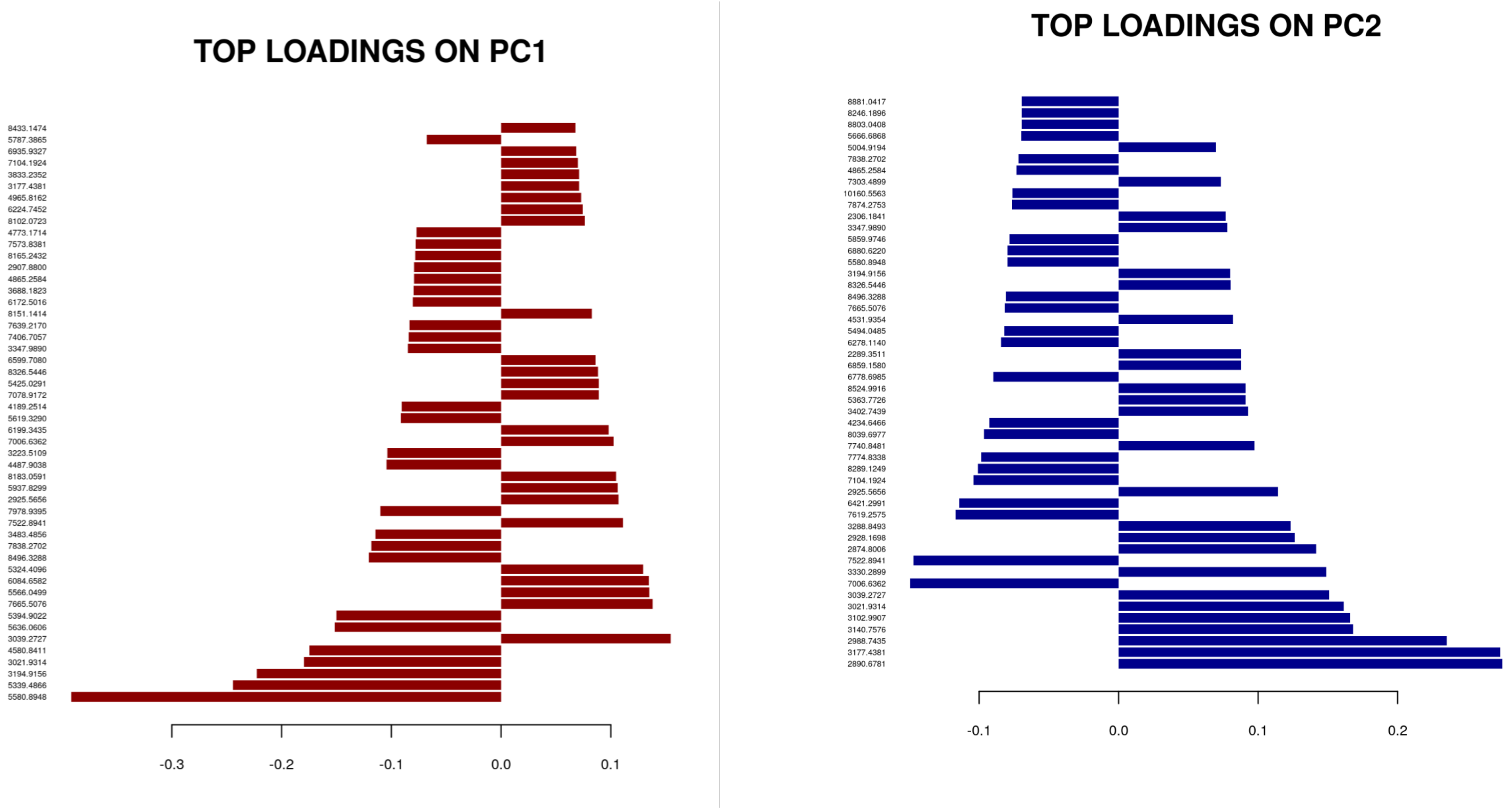

Figure S4. Top loadings on PC1 and PC2 from PCA analysisof albuminextractsfrom certified seedsof Finolaand SecuieniJ. varieties.

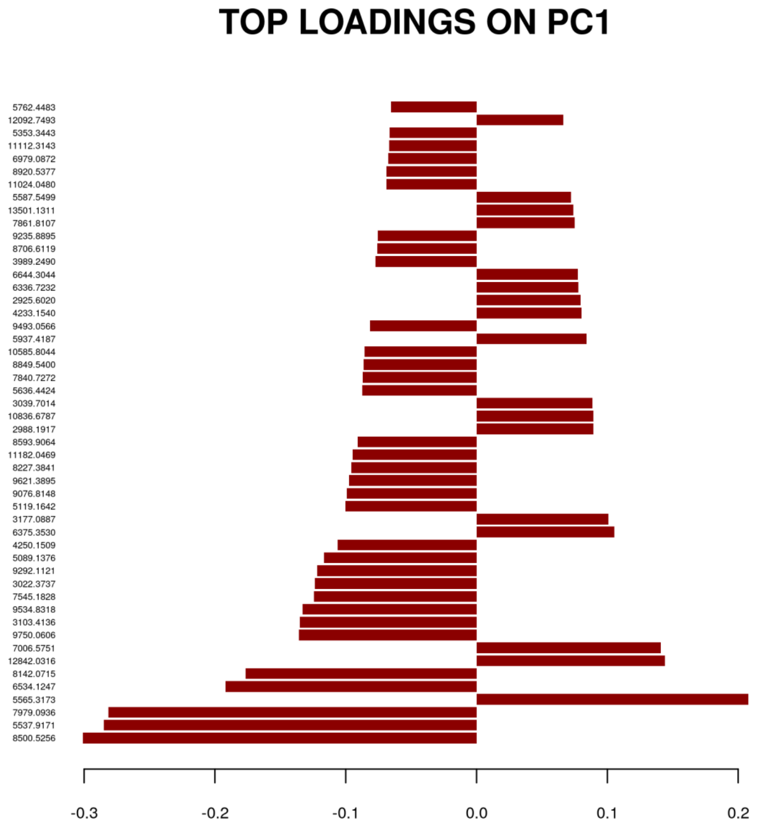

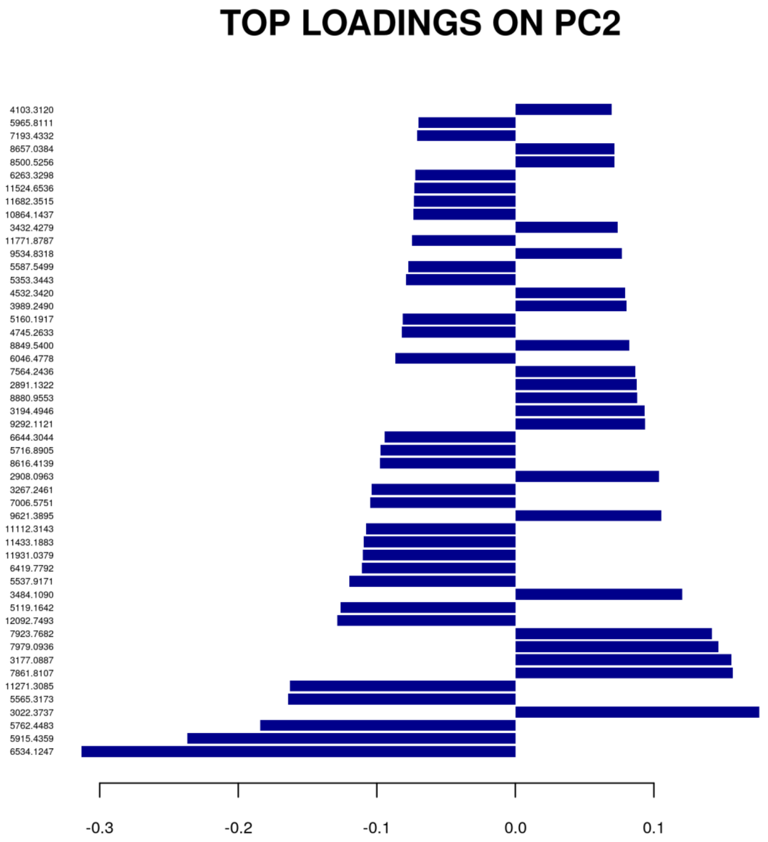

Figure S5. Top loadings on PC1 and PC2 fromPCA analysisof globulinextractsfrom certified seedsof Finolaand SecuieniJ. varieties.

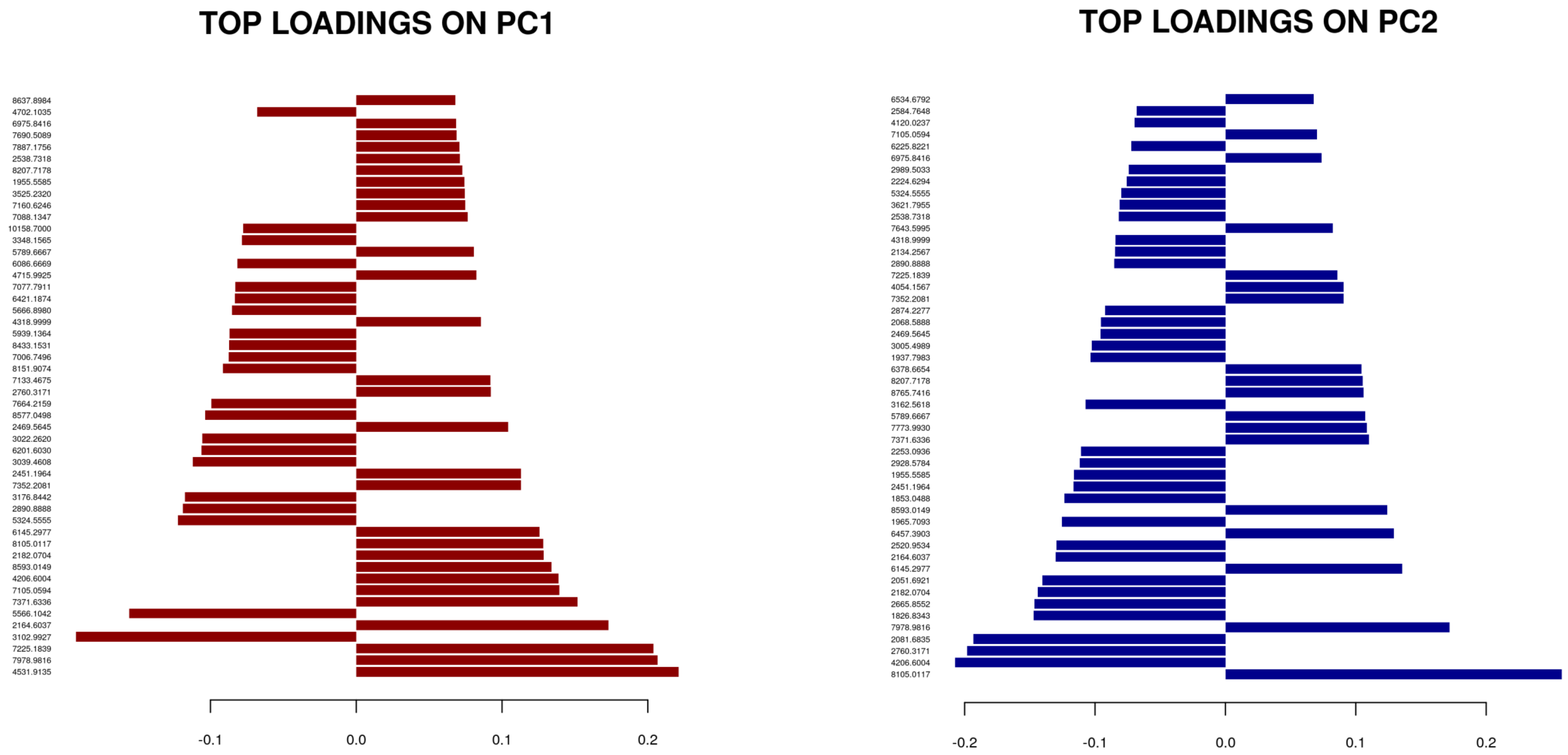

Figure S6. Top loadings on PC1 and PC2 fromPCA analysisof albuminextractsfrom certified and Viganella seedsof Finolavariety.

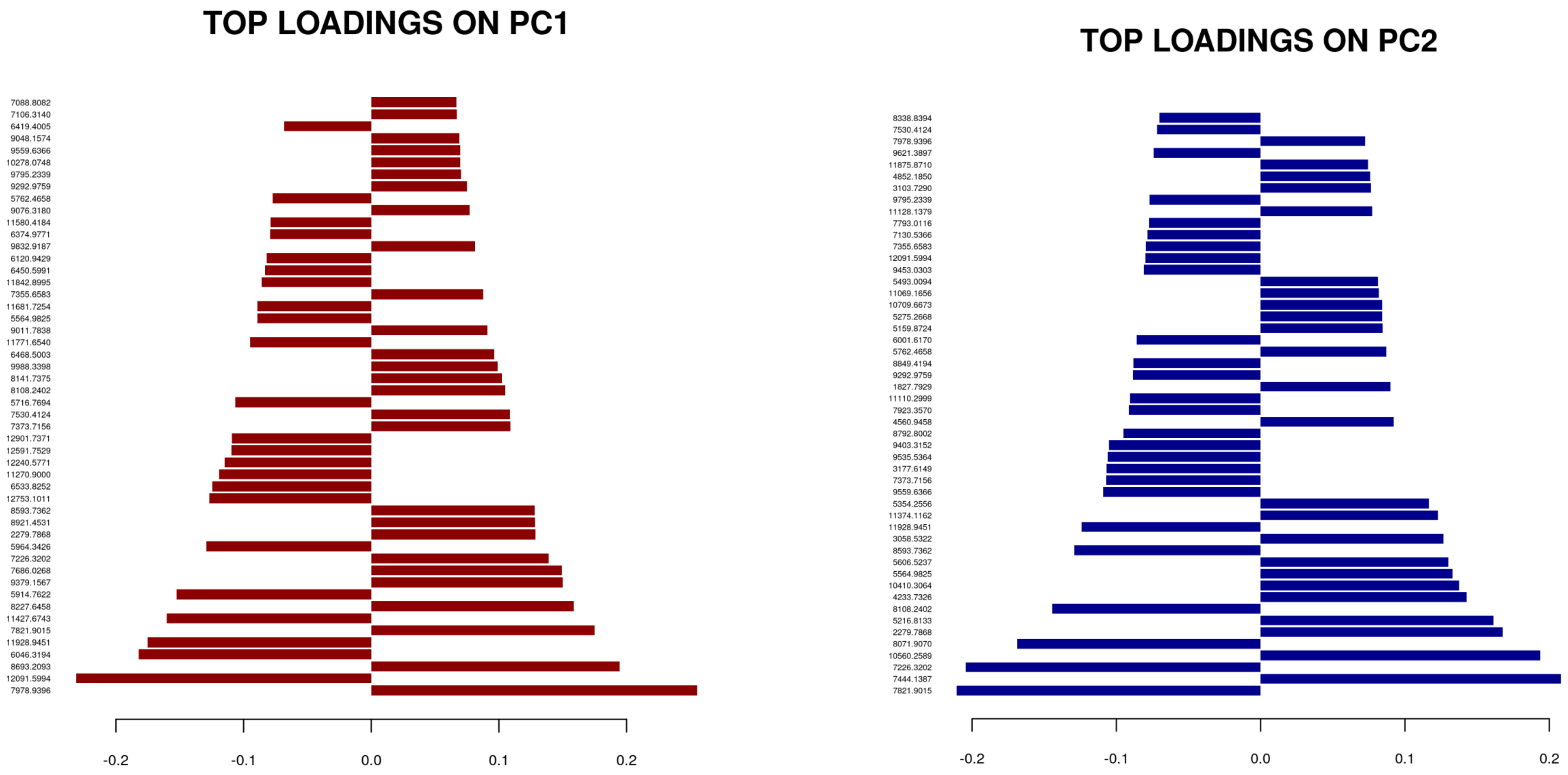

Figure S7. Top loadings on PC1 and PC2 fromPCA analysisof globulinextractsfrom certified and Viganella seedsof Finolavariety.

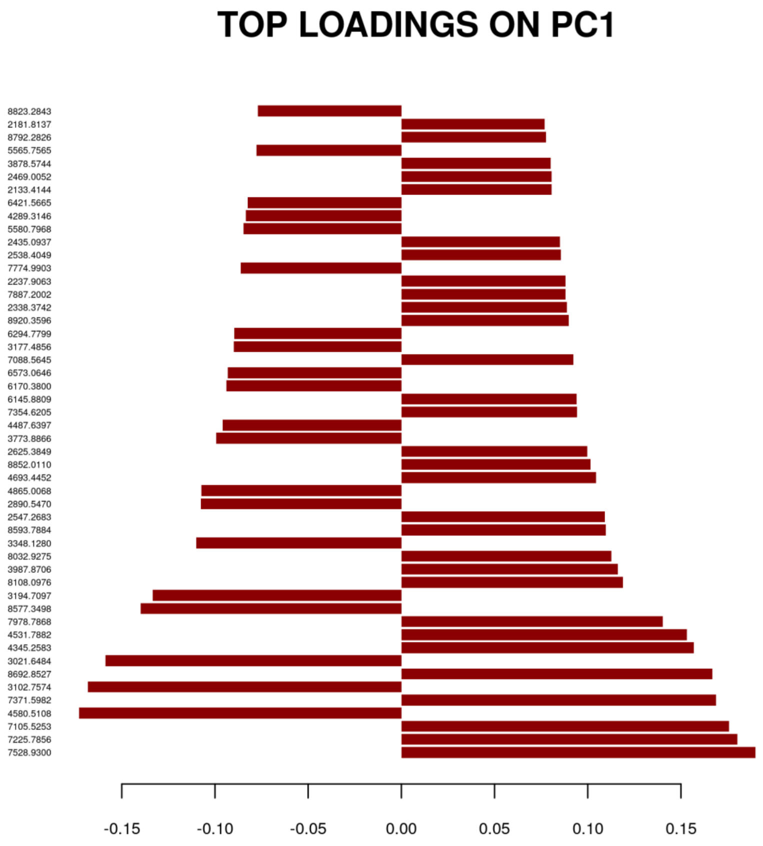

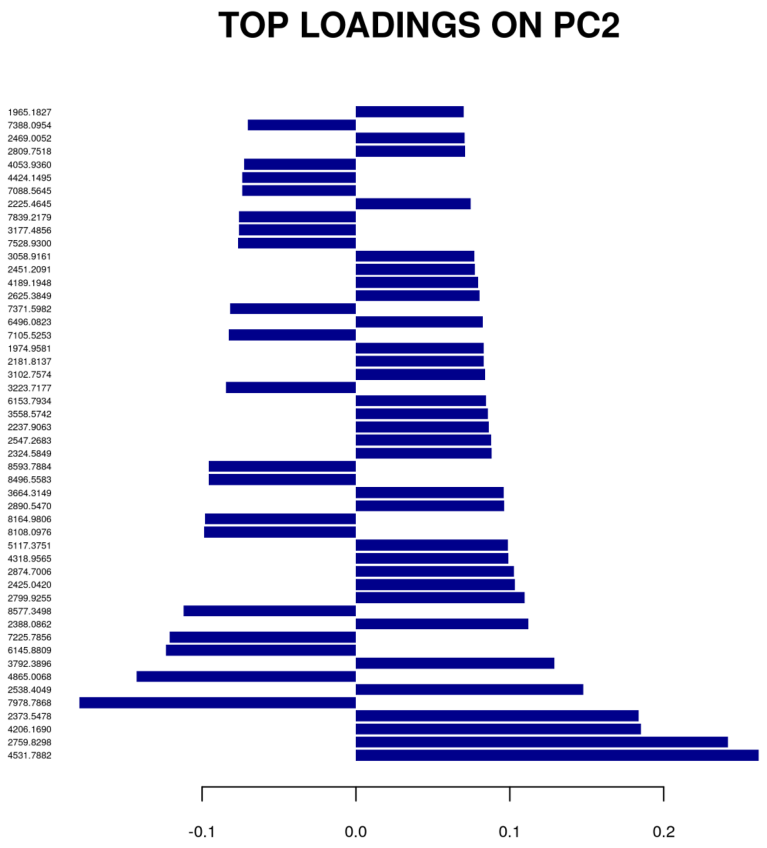

Figure S8. Top loadings on PC1 and PC2 from PCA analysisof albuminextractsfrom certified and Viganella seedsof SecuieniJ. variety.

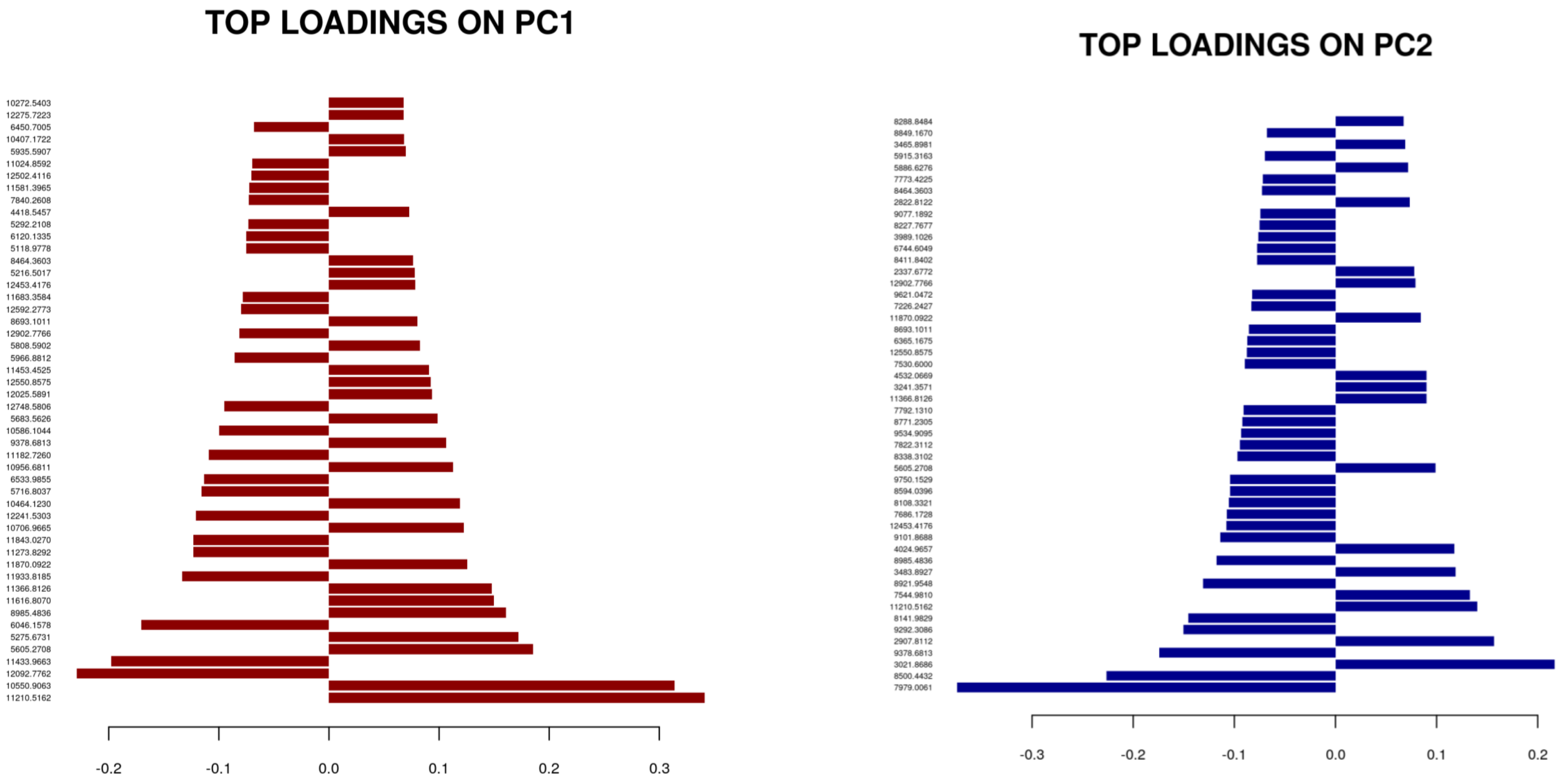

Figure S9. Top loadings on PC1 and PC2 fromPCA analysisof globulinextractsfrom certified and Viganella seedsof SecuieniJ. variety.
